# A bedside to bench study of anti-PD-1, anti-CD40, and anti-CSF1R indicates that more is not necessarily better

**DOI:** 10.1186/s12943-023-01884-x

**Published:** 2023-11-14

**Authors:** Dijana Djureinovic, Sarah A. Weiss, Irina Krykbaeva, Rihao Qu, Ioannis Vathiotis, Myrto Moutafi, Lin Zhang, Ana L. Perdigoto, Wei Wei, Gail Anderson, William Damsky, Michael Hurwitz, Barbara Johnson, David Schoenfeld, Amit Mahajan, Frank Hsu, Kathryn Miller-Jensen, Yuval Kluger, Mario Sznol, Susan M. Kaech, Marcus Bosenberg, Lucia B. Jilaveanu, Harriet M. Kluger

**Affiliations:** 1 Department of Medicine (Medical Oncology), Yale University School of Medicine, 333 Cedar Street, WWW211B, New Haven, CT 06520 USA; 2 Department of Pathology, Yale University School of Medicine, New Haven, CT USA; 3 Department of Internal Medicine, Yale University, New Haven, CT USA; 4 Department of Biostatistics, Yale School of Public Health, New Haven, CT USA; 5 Department of Dermatology, Yale University School of Medicine, New Haven, CT USA; 6 Department of Radiology and Biomedical Imaging, Yale University School of Medicine, New Haven, CT USA; 7 Apexigen, Inc., San Carlos, CA USA; 8 Department of Biomedical Engineering, Yale University, New Haven, CT USA; 9 Department of Molecular, Cellular, and Developmental Biology, Yale University, New Haven, CT USA; 10 Systems Biology Institute, Yale University, New Haven, CT USA; 11 NOMIS Center for Immunobiology and Microbial Pathogenesis, Salk Institute, La Jolla, CA USA; 12 Department of Immunobiology, Yale University School of Medicine, New Haven, CT USA

**Keywords:** CD40, Clinical trial, CSF1R, PD-1, Melanoma, Checkpoint inhibition resistance

## Abstract

**Background:**

Stimulating inflammatory tumor associated macrophages can overcome resistance to PD-(L)1 blockade. We previously conducted a phase I trial of cabiralizumab (anti-CSF1R), sotigalimab (CD40-agonist) and nivolumab. Our current purpose was to study the activity and cellular effects of this three-drug regimen in anti-PD-1-resistant melanoma.

**Methods:**

We employed a Simon’s two-stage design and analyzed circulating immune cells from patients treated with this regimen for treatment-related changes. We assessed various dose levels of anti-CSF1R in murine melanoma models and studied the cellular and molecular effects.

**Results:**

Thirteen patients were enrolled in the first stage. We observed one (7.7%) confirmed and one (7.7%) unconfirmed partial response, 5 patients had stable disease (38.5%) and 6 disease progression (42.6%). We elected not to proceed to the second stage. CyTOF analysis revealed a reduction in non-classical monocytes. Patients with prolonged stable disease or partial response who remained on study for longer had increased markers of antigen presentation after treatment compared to patients whose disease progressed rapidly. In a murine model, higher anti-CSF1R doses resulted in increased tumor growth and worse survival. Using single-cell RNA-sequencing, we identified a suppressive monocyte/macrophage population in murine tumors exposed to higher doses.

**Conclusions:**

Higher anti-CSF1R doses are inferior to lower doses in a preclinical model, inducing a suppressive macrophage population, and potentially explaining the disappointing results observed in patients. While it is impossible to directly infer human doses from murine studies, careful intra-species evaluation can provide important insight. Cabiralizumab dose optimization is necessary for this patient population with limited treatment options.

**Trial registration:**

ClinicalTrials.gov Identifier: NCT03502330.

**Supplementary Information:**

The online version contains supplementary material available at 10.1186/s12943-023-01884-x.

## Background

Immune checkpoint inhibitors targeting programmed cell death protein-1 (PD-1), its ligand (PD-L1), cytotoxic T-lymphocyte associated protein-4 (CTLA-4) or lymphocyte activation gene-3 have improved the survival of patients with multiple cancer types. Objective response rates (ORR) for advanced melanoma or renal cell carcinoma (RCC) patients treated with dual immune checkpoint blockade (anti-CTLA-4 and anti-PD-1) are 57.6% and 42%, respectively [1, 2]. Although durable responses are observed in a subgroup of patients, most patients are resistant to checkpoint inhibition. Novel treatments or combinations are necessary to overcome this resistance and improve durable response rates.

Resistance to checkpoint inhibition has been associated with impaired T-cell function or lack of tumor infiltrating lymphocytes (TILs) in the tumor microenvironment (TME), which can be result of immune suppressive mechanisms caused by tumor associated macrophages (TAMs) [3, 4]. TAMs suppress adaptive immunity by stimulating production of immune suppressive factors such as interleukin-10, transforming growth factor beta, prostaglandin E2, and arginase 1 (ARG1) that inhibit cytotoxic T-cell activity and promote recruitment of regulatory T-cells [5]. TAMs secrete growth factors and cytokines that can support angiogenesis, tumor growth and invasion [6]. Given their potent immunosuppressive properties and critical role in the TME, TAMs have become attractive targets of anti-cancer therapy. Preclinical studies performed by our group and others have demonstrated potential activity for TAM modulation using a colony-stimulating factor 1 receptor (CSF1R) blocking antibody (αCSF1R) and a CD40 agonist (CD40a) [7–9]. CSF1 is produced by various types of mesenchymal and epithelial cells and is a key regulator of monocytes and macrophages [10, 11]. One of its receptors, CSF1R, is expressed by macrophages, DCs, neutrophils, myeloid-derived suppressor cells (MDSCs), and granulocytes [12]. Blocking CSF1R signaling decreases the recruitment of TAMs and reduces tumor growth in several tumor models by elimination or repolarization of TAMs [3, 13, 14]. Small molecule inhibitors and monoclonal antibodies against CSF1 or CSF1R have been assessed in early phase studies, most commonly as monotherapy or in combination with immunotherapy [15]. Except for tenosynovial giant cell tumors, activity of drugs targeting CSF1/CSF1R has been disappointing so far in patients with advanced cancers. Finding the optimal combination partner(s) to pair with CSF1/CSF1R blockade and establishing the most effective dose is paramount.

One promising approach that involves modulation of macrophages, along with other pro-inflammatory cells, is through activation of the CD40 receptor by the CD40 ligand or agonistic antibodies [16]. CD40 is a costimulatory receptor molecule that is mainly expressed on antigen presenting cells. Activation of CD40 stimulates tumor-specific antigen presentation, resulting in cytotoxic T-cell recruitment, and secretion of pro-inflammatory cytokines by macrophages that support their tumoricidal activity. Studies evaluating combined CSF1R blockade and CD40a in humans are limited [17]. Preclinical studies by our group have suggested improved activity of αCSF1R and CD40a when combined with αPD-1 compared to doublet therapies (unpublished data). Based on these preclinical data we conducted a phase I/Ib trial of patients with advanced melanoma, RCC or non-small cell lung cancer (NSCLC) resistant to αPD-(L)1 to determine the safety and recommended phase 2 dose (RP2D) of αCSF1R (cabiralizumab) combined with CD40a (sotigalimab) with or without αPD-1 (nivolumab) [18]. In the phase I dose escalation part of the trial, the triplet regimen was safe, with upregulation of pro-inflammatory cytokines and chemokines, and the RP2D of the triplet was established [18]. Here we report the clinical outcomes of the phase Ib melanoma dose expansion portion of the trial, studying the RP2D of the triplet combination in patients with advanced melanoma resistant to αPD-(L)1 and the pharmacodynamic studies from the phase Ib component. Due to limited activity of the triplet combination, we examined dosing implications for the αCSF1R and reverted to preclinical models to understand the mechanisms whereby this regimen could be better optimized in this patient population.

## Results

### Insufficient antitumor activity of phase Ib

Between February 2019 and November 2019, 13 patients were enrolled in the first stage of the melanoma disease-specific phase Ib at the triplet therapy (nivolumab, cabiralizumab, and sotigalimab) RP2D from February 2019 to November 2021. Table 1 displays baseline patient characteristics.

**Table 1:** Baseline characteristics of melanoma patients on phase Ib portion

**Age**	
Median	65
Range	55–84
**Sex**	
Male	7 (54%)
Female	6 (46%)
**Race/Ethnicity**	
White Non-Hispanic	11 (84.6%)
White Hispanic or Latino	1 (7.7%)
Black or African American	1 (7.7%)
**ECOG Performance Status**	
0	7 (54%)
1	6 (46%)
**BRAF status**	
V600E/K mutated	4 (31%)
WT	9 (69%)
**LDH**	
≤ Upper limit of normal	7 (54%)
> Upper limit of normal	6 (46%)
**All lines of prior treatment**	
Median	2
Range	1–6
**Lines of prior immunotherapy-based treatment only**	
Median	2
Range	1–4
**Prior Ipi + Nivo**	
	11 (85%)

**Fig. 1:**
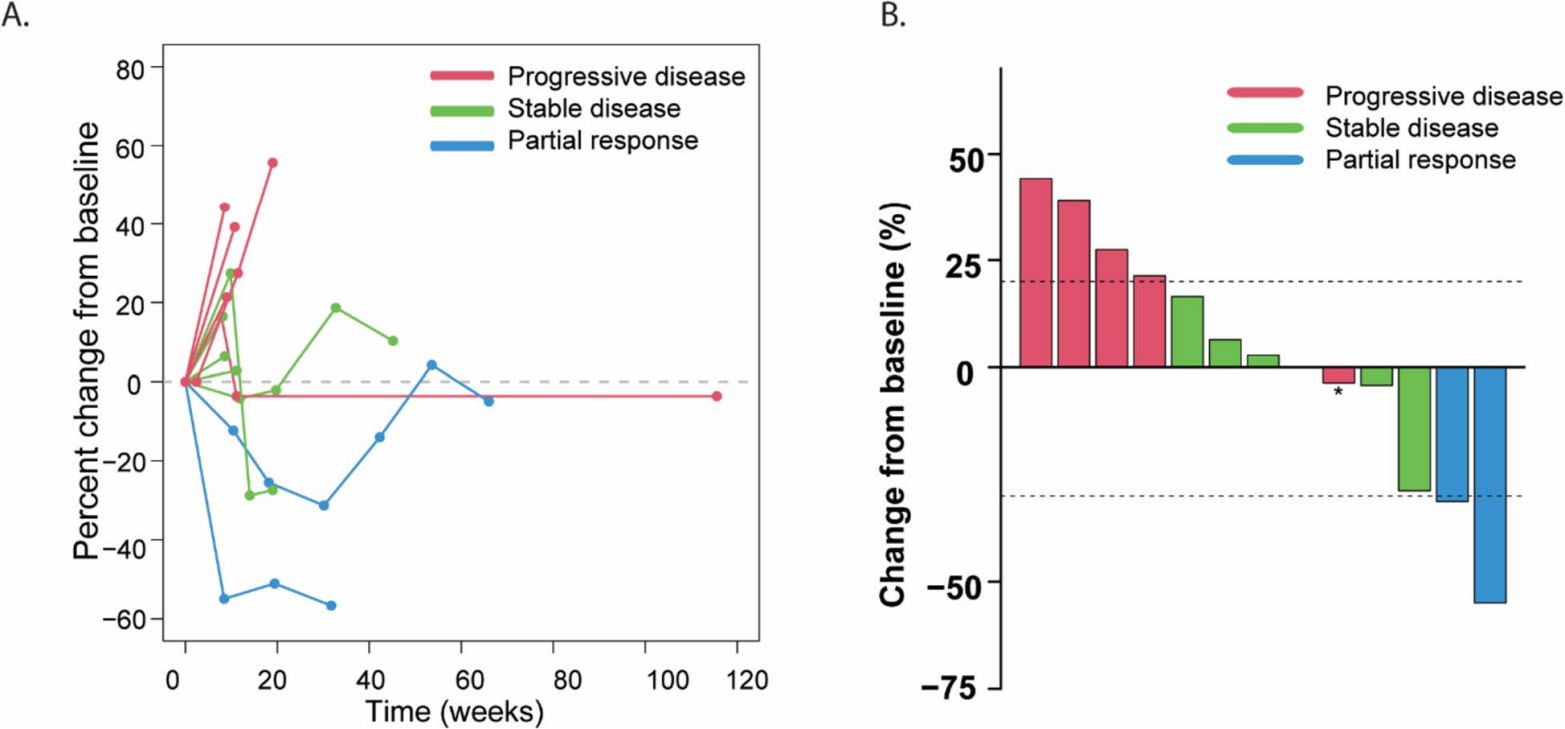
Response of 13 melanoma patients on phase Ib **A**. Spider plot showing percent change from baseline over time. **B** Best overall response for each patient depicted by waterfall plot. Asterisk denotes progression in patient in non-target lesions

Patients were on study for a median of 2.4 months, ranging from 1.9 to 14.8 months. Median follow-up time was 3.7 months. Data cut-off was January 31, 2022. ORR was 7.7% with one confirmed partial response (PR) and 1 unconfirmed PR. Five patients had stable disease (SD) as the best response (38.5%) and 6 had progressive disease (PD) (46.2%) (Table 2, Fig. 1A and B). Because there were less than 2 confirmed PRs in the first 13 patients enrolled, the study did not proceed to the second stage. The patient with a confirmed PR had a normal lactate dehydrogenase and Eastern Cooperative Oncology Group (ECOG) performance status of 0 at baseline. His only prior line of therapy was ipilimumab plus nivolumab. This patient remained on study for 6.8 months at which point his disease had progressed. Median progression free survival (PFS) was 2.4 months. Median overall survival (OS) from time of initiation of therapy was 10.5 months, Table 2.

**Table 2:** Best overall response

**Best Overall Response**	*n* = 13 (%)
Partial Response (confirmed)	1 (7.7)
Partial Response (unconfirmed)	1 (7.7)
Stable Disease	5 (38.5)
Progressive Disease	6 (46.2)
**Objective Response Rate**	7.7%
**Disease Control Rate**	46.2%

### Safety (phase Ib)

Adverse events and serious adverse events for 13 patients were graded per the National Cancer Institute Common Terminology Criteria for Adverse Events v5.0 from treatment initiation until 100 days after treatment or until a new anti-cancer therapy began (Table 3). Treatment-related adverse events (TRAEs) occurred in 100% of patients. The majority of TRAEs were grade 1–2 consisting of asymptomatic laboratory abnormalities including aminotransferase and creatine phosphokinase (CPK) elevations, periorbital edema, fatigue, and transient symptoms related to sotigalimab infusion including fever and chills. Grade 3–4 TRAEs were uncommon and mostly consisted of asymptomatic laboratory value abnormalities. These included grade 3 elevations of aspartate aminotransferase (AST) (*n* = 4), CPK (*n* = 4), and bilirubin (*n* = 1), and grade 4 elevations of AST (*n* = 1) and alanine aminotransferase (*n* = 1).

**Table 3:** Treatment-related adverse events

*N* = 13	**Related to nivolumab alone**		**Related to cabiralizumab alone**		**Related to sotigalimab alone**		**Related to all**	
Treatment-Related Adverse Events	Grade 1–2	Grade ≥ 3	Grade 1–2	Grade ≥ 3	Grade 1–2	Grade ≥ 3	Grade 1–2	Grade ≥ 3
	n (%)	n (%)	n (%)	n (%)	n (%)	n (%)	n (%)	n (%)
Chills	-	-	-	-	8 (62)	0 (0)	-	-
Fatigue & weakness	1 (8)	0 (0)	-	-	-	-	8 (62)	0 (0)
Periorbital edema	-	-	6 (46)	0 (0)	-	-	-	-
Alanine aminotransferase increase	-	-	-	-	-	-	7 (54)	1 (8)
Aspartate aminotransferase increase	-	-	-	-	-	-	3 (23)	5 (38)
Creatine phosphokinase increase	-	-	2 (15)	4 (31)	-	-	-	-
Fever	-	-	-	-	4 (31)	0 (0)	-	-
Nausea/vomiting	-	-	-	-	3 (23)	0 (0)	-	-
Diarrhea	1 (8)	0 (0)	-	-	-	-	-	-
Headache	-	-	-	-	3 (23)	0 (0)	-	-
Rash—maculopapular	-	-	-	-	-	-	3 (23)	0 (0)
Hypothyroidism	2 (15)	0 (0)	-	-	-	-	-	-
Creatinine increase	-	-	-	-	-	-	2 (15)	0 (0)
Pruritis	-	-	-	-	-	-	1 (8)	0 (0)
Myalgia	-	-	-	-	1 (8)	0 (0)	-	-
Lipase increase	1 (8)	0 (0)	-	-	-	-	-	-
Thrush	1 (8)	0 (0)	-	-	-	-	-	-
Hypotension	-	-	-	-	1 (8)	0 (0)	-	-
Hypoglycemia	-	-	-	-	-	-	0 (0)	1 (8)
Flushing	-	-	-	-	1 (8)	0 (0)	-	-
Edema in limbs	-	-	1 (8)	0 (0)	-	-	-	-
Bilirubin increase	-	-	-	-	-	-	0 (0)	1 (8)
Sinus tachycardia	-	-	-	-	1 (8)	0 (0)	-	-
Anemia	-	-	-	-	-	-	1 (8)	0 (0)
Platelet count decrease	-	-	-	-	-	-	1 (8)*	0 (0)

^*^attributed to nivolumab and sotigalimab only

### Reduction in non-classical monocytes and B-cells in humans after treatment

**Fig. 2:**
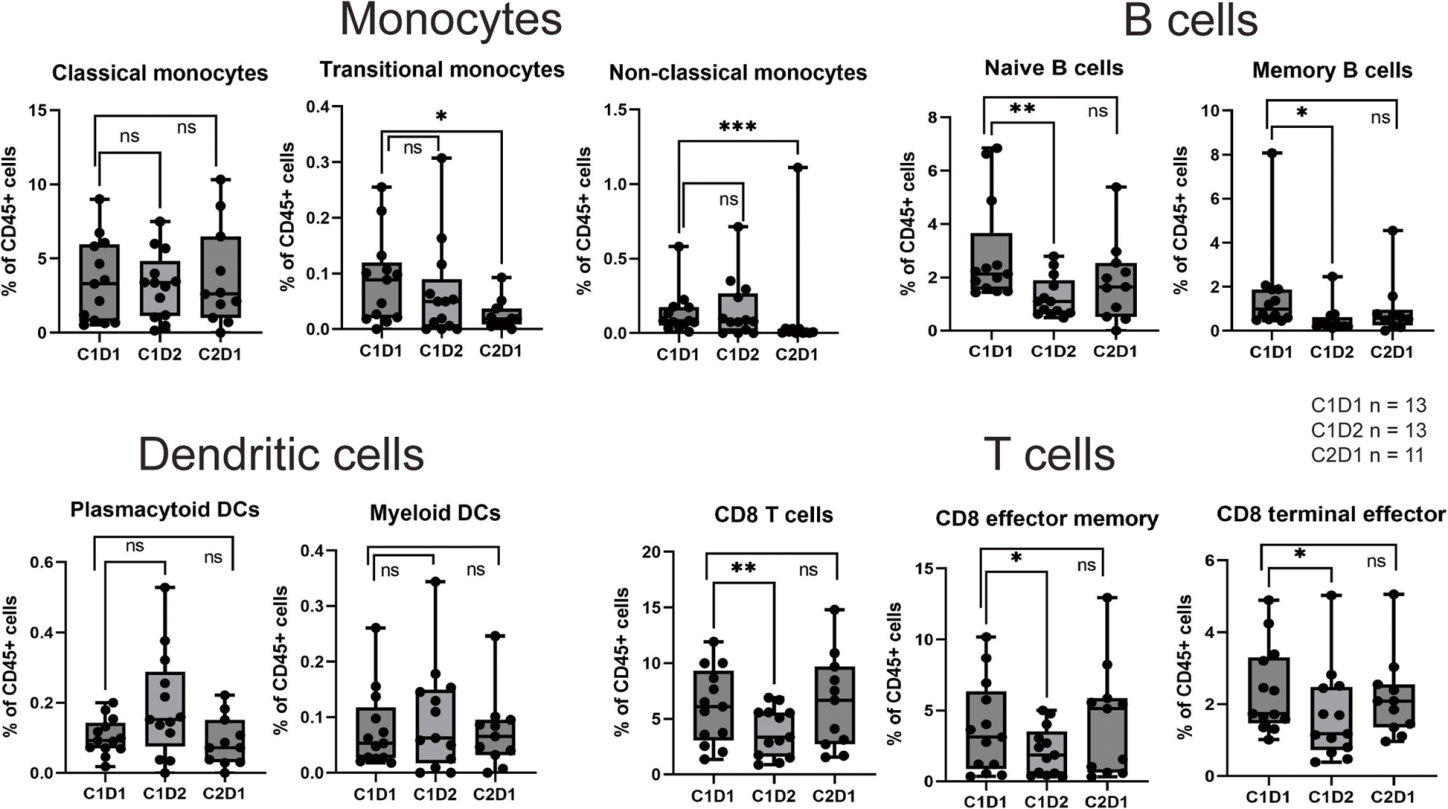
Changes in peripheral blood mononuclear cell (PBMC) populations. Percentage of indicated cell type in patients treated with sotigalimab, cabiralizumab and nivolumab at cycle 1, day 1 (C1D1), cycle 1, day 2 (C1D2) and cycle 2, day 1 (C2D1). ns = not significant, *p < 0.05, **p < 0.01, ***p < 0.001

To analyze treatment related changes in circulating immune cells, cytometry by time of flight (CyTOF) analysis was performed on peripheral blood mononuclear cells (PBMCs) from 13 patients from the phase Ib component drawn before treatment on cycle 1, day 1 (C1D1), 24 h after treatment on cycle 1, day 2 (C1D2) and before treatment on cycle 2, day 1 (C2D1). The proportions of different cell populations were compared between baseline and the two timepoints on treatment in all patients. Non-classical monocytes (CD38^−^CD14^−^) and transitional monocytes (CD38^lo^CD14^int^) were significantly reduced at C2D1 (*p* < 0.05 for both). There was a trend towards an increase in plasmacytoid DCs at C1D2 (*p* = 0.06). A significant reduction of both naïve-and memory B-cells was observed at C1D2 (*p* =  < 0.05 for both). Among T-cell populations, total CD8 T-cells, CD8 effector memory cells and CD8 terminal effector cells were significantly reduced at C1D2 (*p* = 0.03 for all) (Fig. 2). No significant changes were observed in other T-cell populations, NK cells or granulocytes (Supplementary Fig. 1).

### Associations between changes in human PBMCs and patient characteristics

**Fig. 3:**
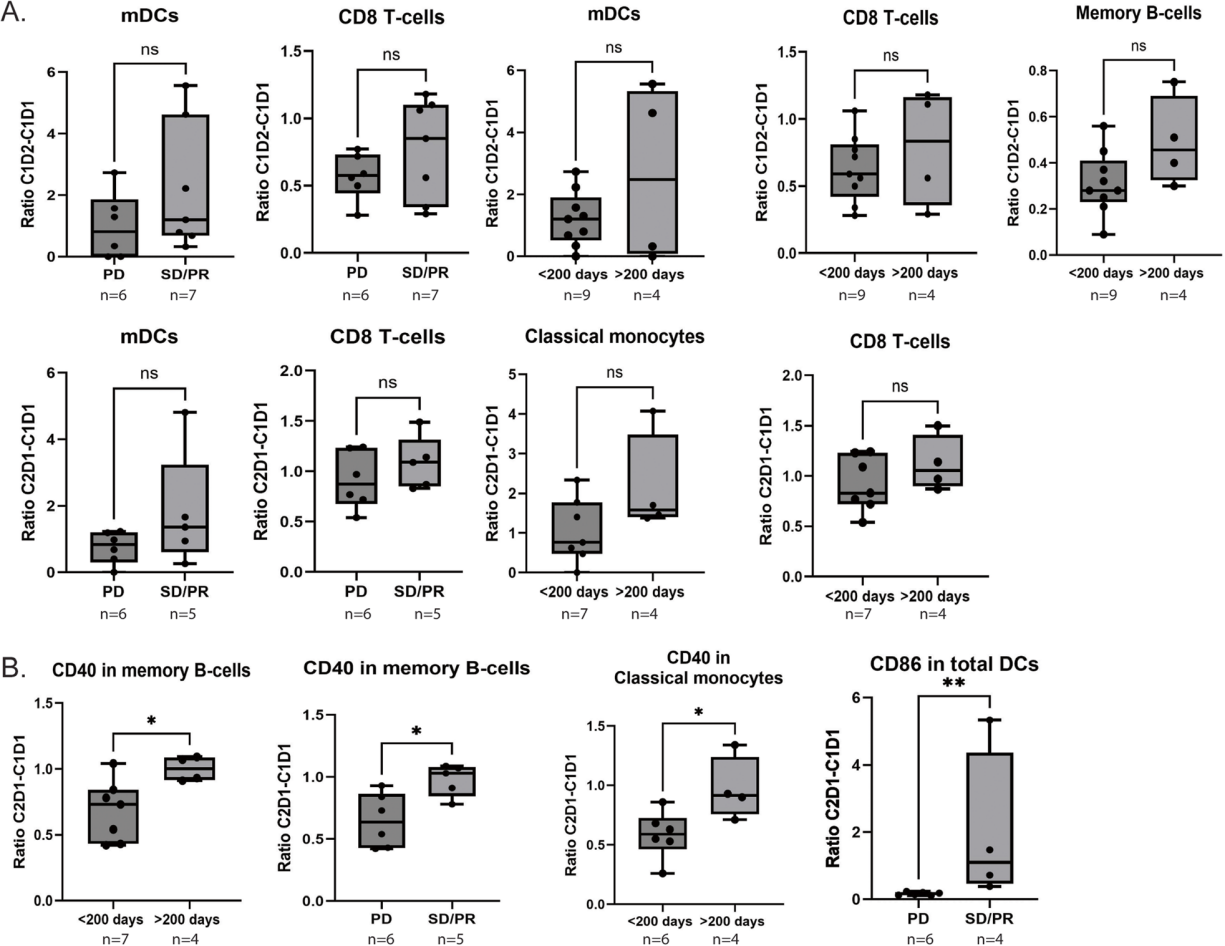
Associations between clinical benefit and changes in human cell populations or individual markers. A. Ratio of percentages of myeloid DCs, CD8 T-cells, memory B-cells and classical monocytes at C1D2 over C1D1 or C2D1 over C1D1. B. Ratio of average expression of CD40 or CD86 at C2D1 over C1D1 in indicated cell types in patients with progressive disease versus stable/partial response and in short-term (< 200 days) versus prolonged (> 200 days) trial duration

The ratios of different cell populations between C1D1-C1D2 and C1D1-C2D1 were analyzed for changes associated with best response and duration of time on trial, as a measure of possible clinical benefit. Patients with stable disease or partial response had an increase in myeloid DCs and CD8 T-cells at both C1D2 and C2D1 relative to baseline compared to patients with progressive disease. An increase in myeloid DCs and memory B-cells at C1D2, classical monocytes at C2D1 and CD8 T-cells at both timepoints was observed in patients that had been on trial for over 200 days compared to patients that had been on trial for less than 200 days. None of the changes met statistical significance (Fig. 3a). An increase of CD40 in B cells and classical monocytes and CD86 in DCs was associated with better clinical outcome (Fig. 3b). There was a trend towards increased expression of CD86 (*p* = 0.07) and PD-L1 (*p* = 0.07) in classical monocytes in patients that had been longer on trial (data not shown).

### Higher αCSF1R dose increased tumor growth and decreased survival in the YUMMER1.7 model

**Fig. 4:**
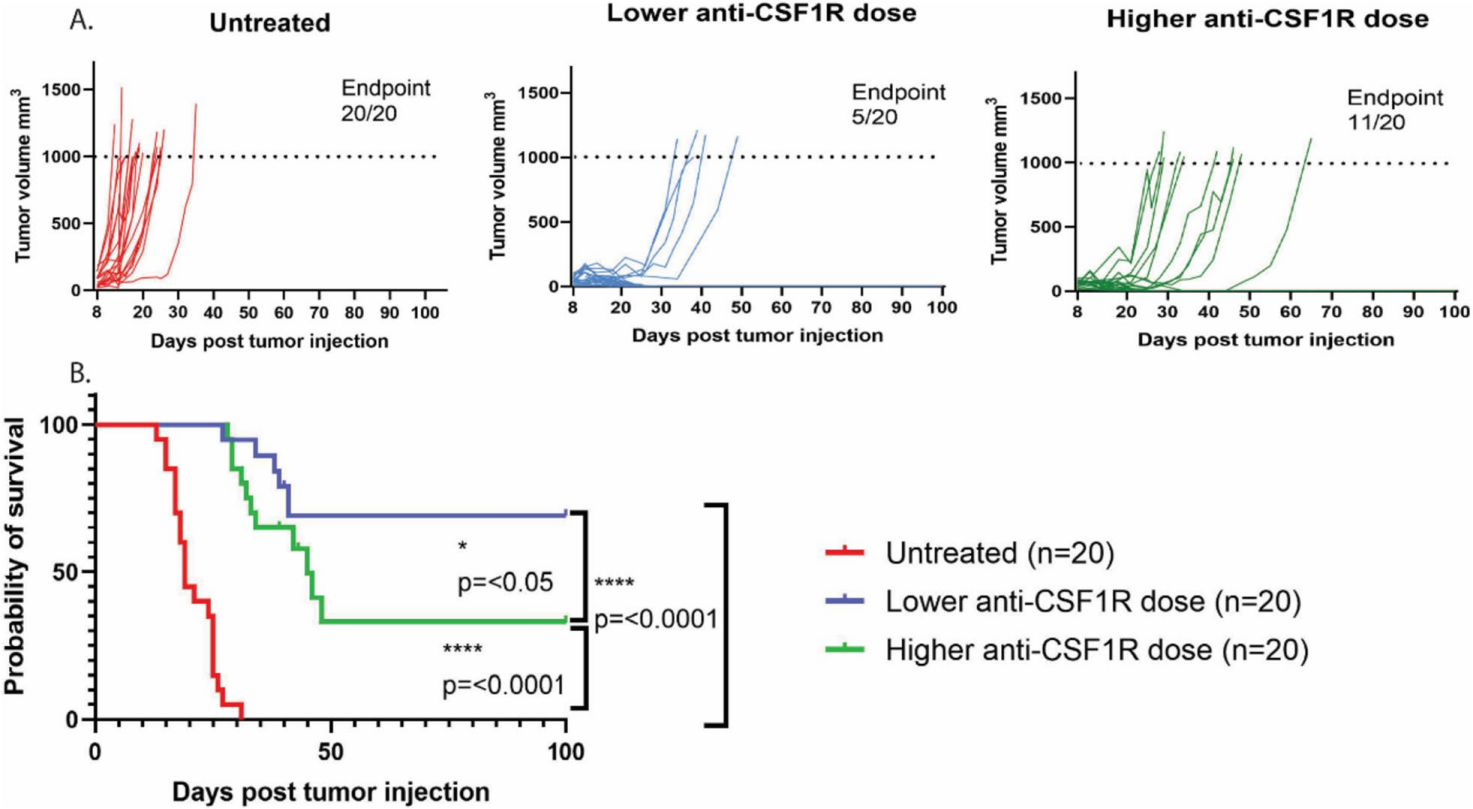
Higher dose of anti-CSF1R increases tumor growth and decreases survival in YUMMER1.7 melanoma model. A. Tumor volumes in untreated YUMMER1.7 melanoma mice or YUMMER1.7 melanoma mice treated with anti-PD-1 + anti-CD40 with either low (200ug) or high (400ug) dose of anti-CSF1R. B. Survival curve of untreated YUMMER1.7 melanoma mice and YUMMER1.7 melanoma mice treated with anti-PD-1 + anti-CD40 with either low (200ug) or high (400ug) dose of anti-CSF1R * *p* =  < 0.05, **** *p* =  < 0.0001

Sotigalimab, cabiralizumab and nivolumab eliminated non-classical monocytes and increased antigen presenting cells but did not translate into meaningful clinical activity in humans. Due to the limited activity in humans and our previous studies in mice supporting evaluation of the triplet in humans, we tested αPD-1, CD40a and αCSF1R dosing implications in YUMMER1.7 (Yale University Mouse Melanoma Exposed to Radiation 1.7). YUMMER1.7 is an immune competent melanoma model, relatively resistant to immunotherapy with anti-PD1 + anti-CTLA4 as the majority of YUMMER1.7-bearing mice have tumor progression on anti-PD1 monotherapy and do not reject their tumors [19]. Seven days after tumor cell injection, ten mice were treated with αPD-1, CD40a and lower αCSF1R dose while a second group received αPD-1, CD40a and higher αCSF1R dose. Ten mice were treated with PBS. The entire experiment was repeated with 10 additional mice per cohort, for a total of 20 mice per cohort. Tumors in all control treated mice (20/20) reached the endpoint of 1000 mm^3^; in the group treated with higher dose αCSF1R, 55% of mice (11/20) reached endpoint, whereas mice treated with lower dose αCSF1R fared better with 25% (5/20) reaching the endpoint (Fig. 4a). The treatment combinaton with lower αCSF1R dose resulted in improved survival compared to the higher αCSF1R dose (p = 0.048) (Fig. 4b). We repeated the analysis in the parental cell line YUMM1.7 which has same three driver mutations *Braf*^V600E^, Pten ^−/−^, and *Cdkn2a*^−/−^ but a lower number of somatic mutations and is not immunogenic at all [19]. Treatment with both lower and higher anti-CSF1R dose delayed the tumor growth compared to untreated mice but all tumors eventually grew out to endpoint and there was no difference in survival between the two treatment groups. In YUMM1.7 tumors, the lower anti-CSF1R dose was as ineffective as the higher anti-CSF1R dose (Supplementary Fig. 2).

### YUMMER1.7 tumors treated with higher αCSF1R dose have more CD163 + cells

**Fig. 5:**
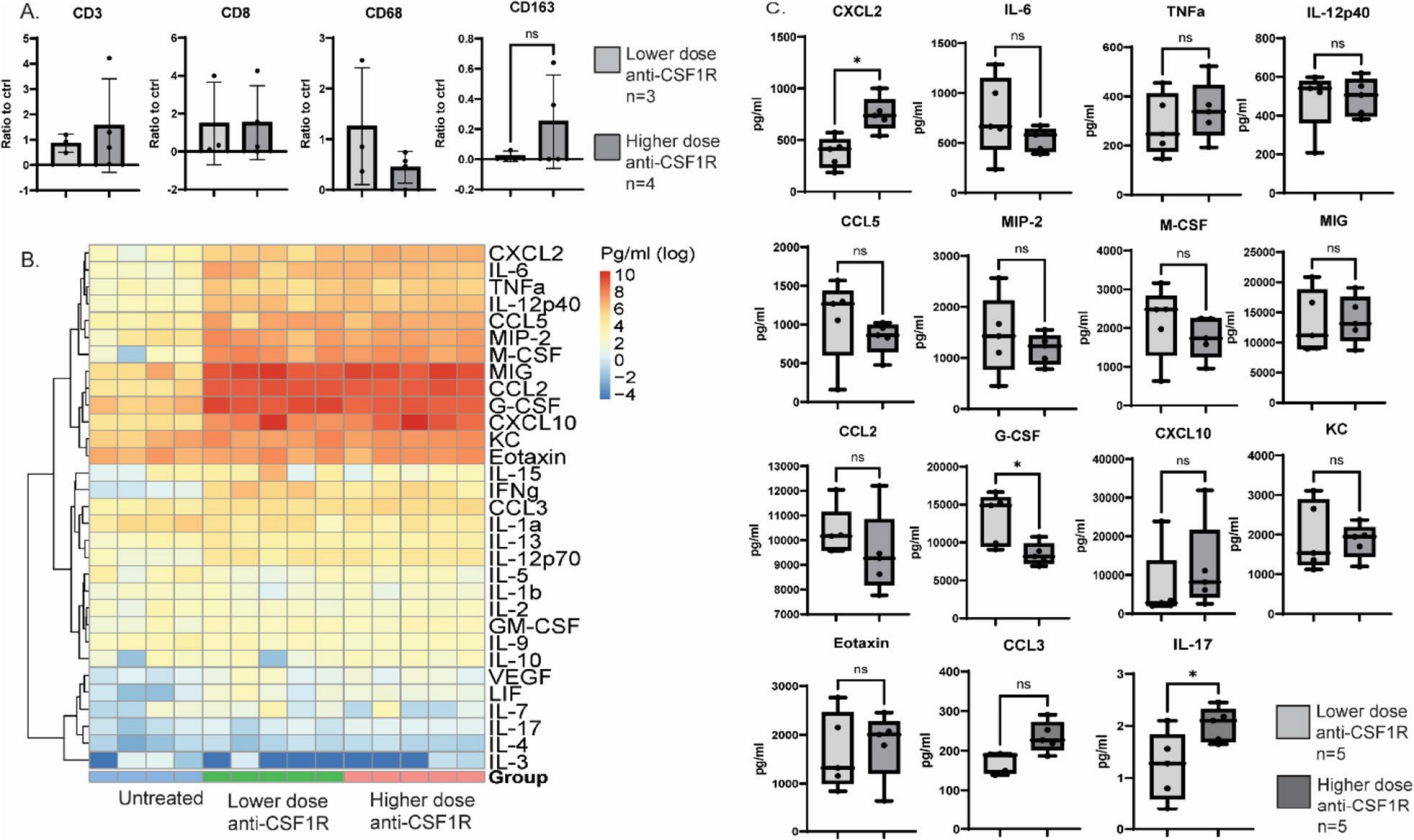
Effects of lower vs. higher anti-CSF1R doses on immune cell infiltrates and circulating cytokines and chemokines in the YUMMER1.7 model. A. YUMMER1.7 endpoint tumor tissue positivity of CD3, CD8, CD68 and CD163 from mice that received a lower anti-CSF1R dose + anti-CD40 and anti-PD-1 and higher anti-CSF1R dose together with anti-CD40 and anti-PD-1 displayed as ratio to control (untreated) tumors. B. Cytokine/ chemokine profiling in YUMMER1.7 mice 24 h after one treatment with PBS (untreated), lower anti-CSF1R dose + anti-CD40 and anti-PD-1 and higher anti-CSF1R dose + anti-CD40 and anti-PD-1. C. Cytokine/chemokine levels in plasma of mice treated with lower anti-CSF1R dose + anti-CD40 and anti-PD-1 and higher anti-CSF1R dose + anti-CD40 and anti-PD-1 collected 24 h post one treatment. ^*^p < 0.05

YUMMER1.7 tumor tissue was collected at endpoint and used for immunohistochemical staining to analyze CD3, CD8, CD68 and CD163 positive cells in the tumor. CD163 staining was used as a marker of pro-tumoral TAM. Positive cells for each marker were analyzed as a ratio to the control tumors. No significant changes were observed. Mice treated with higher αCSF1R dose had more CD163 + cells compared to the group that received lower αCSF1R dose, but the differences did not reach statistical significance (Fig. 5a).

### Effects of lower vs. higher αCSF1R doses on circulating cytokines and chemokines in the YUMMER1.7 model

Treatment related changes in 31 cytokine and chemokine levels were assessed 24 h after intraperitoneal (IP) injection of PBS, lower dose αCSF1R + CD40a + αPD-1 and higher dose αCSF1R + CD40a + αPD-1. Changes were assessed relative to control. Cytokines and chemokines that attract and activate monocytes/macrophages (CXCL10, CCL2, MIG, CCL5, CXCL2, TNFa, and M-CSF) and increase T-cell activation (CCL5, IL12p40, IFNg, and IL15) were increased after treatment compared to control, irrespective of αCSF1R dose (Fig. 5b). Cytokines and chemokines involved in recruitment of neutrophils (G-CSF and KC) and eosinophils (eotaxin) also increased after treatment compared to control, irrespective of αCSF1R dose [20–22]. In the comparison between the treated groups, mice treated with higher αCSF1R dose had higher levels of circulating CXCL2 (*p* = 0.02) and a trend towards increased CCL3 (*p* = 0.06) compared to the group treated with lower αCSF1R dose (Fig. 5c). CXCL2 and CCL3 are monocyte attracting chemokines. Increased levels of CXCL2 and CCL3 have been associated with increased immunesuppressive cells including regulatory T-cells, TAMs and MDSCs [23]. IL-17 was significantly increased in the group that got higher αCSF1R dose (*p* < 0.05). IL-17 is produced by T-cells, recruits neutrophils and promotes the Th2 T-cell type (Fig. 5c) [24]. Granulocyte colony-stimulating factor (G-CSF) was significantly decreased in the group that got higher αCSF1R dose (*p* < 0.05). G-CSF is involved in proliferation and differentiation of myeloid progenitor cells into neutrophils and promotes the mobilization of neutrophils from bone marrow into the blood stream. G-CSF is also involved in the development of immune suppressive macrophages, MDSCs as well as regulatory T-cells (Fig. 5c) [25].

### Higher αCSF1R dose is associated with an immune suppressive gene expression profile of TAMs in YUMMER1.7

**Fig. 6:**
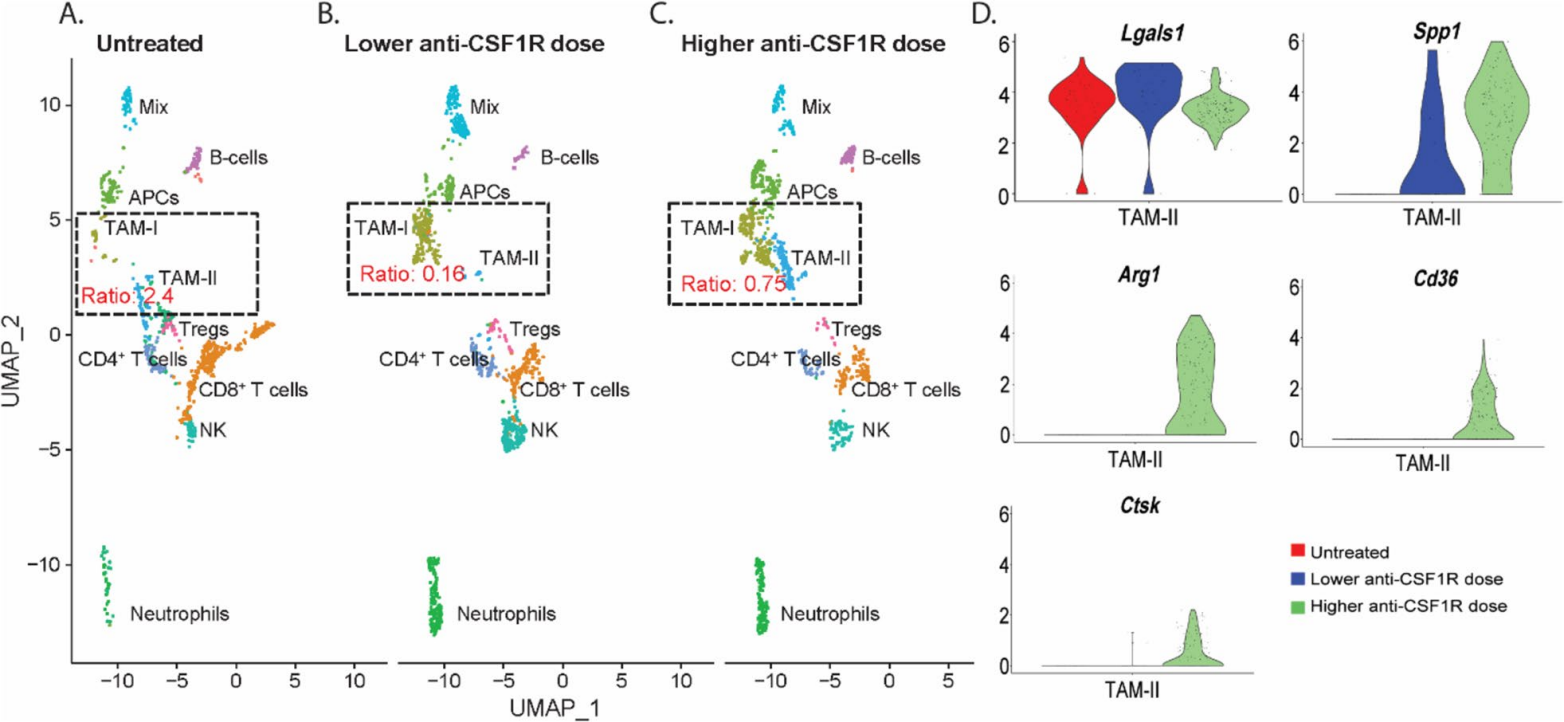
Single cell RNA sequencing analysis of YUMMER1.7 shows higher proportion of TAM-II associated with higher anti-CSF1R dose. A. UMAP visualization of cell populations based on gene expression in YUMMER1.7 24 h after no treatment, B. one treatment with lower anti-CSF1R dose + anti-CD40 and anti-PD-1, C. or higher anti-CSF1R dose together with anti-CD40 and anti-PD-1. D. Gene expression of *Lgals*, *Spp1*, *Arg1*, *Cd36* and *Ctsk* in TAM-II of mice treated as indicated. TAM-I = TAM expressing genes involved in both proinflammatory and suppressive functions. TAM-II = TAM expressing genes involved in predominantly suppressive functions

Subcutaneous YUMMER1.7 tumor tissue was collected from mice 24 h after one treatment. Single cell RNA sequencing (scRNAseq) was performed to analyze differences in gene expression related to αCSF1R dosing. Consistent with the cytokine data showing an increase in cytokines involved in recruitment of neutrophils (G-CSF and KC) in treated mice irrespective of the anti-CSF1R dose, both treatment groups exhibited an increase in neutrophil related genes in the scRNAseq analysis (Fig. 6a-c). Clustering analysis identified two separate populations of TAM. TAM-I cells expressed genes predominantly related to inflammatory function. TAM-II was characterized by genes predominantly related to immune suppression (Supplementary Table 1). The TAM-II populatation was more prevalent in the group that received higher dose of αCSF1R (Fig. 6c) than in the group that recieved lower dose αCSF1R (Fig. 6b). Examples of genes expressed in TAM–II include Lgals1, CD36, Ctsk, SPP1 and Arg1; their respective gene expression in the three treatment conditions is shown in Fig. 6d. Expression of both CCL3 and CXCL2 was increased in the immune suppressive macrophage subset TAM-II in mice that got the higher dose of anti-CSF1R in combination with anti-PD-1 and the CD40a compared to mice that got the lower anti-CSF1R dose (Supplementary Fig. 3).

## Discussion

Here we report clinical and correlative biomarker studies from a phase I/Ib trial of the αCSF1R antibody cabiralizumab in combination with a CD40a sotigalimab and nivolumab in patients with αPD-(L)1-resistant tumors. Other trials with cabiralizumab have been disappointing; despite depletion of non-classical monocytes in blood and TAMs in tissues, limited anti-tumor activity was observed in humans [26]. The rationale for combining CSF1R blockade with CD40a as a strategy to overcome resistance to checkpoint inhibition was motivated by preclinical studies in which this combination fostered a proinflammatory tumor milieu, T-cell stimulation, and TAM depletion [8, 9]. We utilized the RP2D of cabiralizumab, and in a murine model we showed that a lower dose of cabiralizumab might have been more beneficial.

In the first stage of the phase Ib component of the trial in patients with αPD-(L)1-resistant melanoma, limited activity was observed. One patient had a confirmed PR (7.7%), one unconfirmed PR (7.7%), while five patients had SD (38.5%). The study did not proceed to the second stage. Besides the initial phase I dose escalation study [18] and the phase Ib melanoma-specific expansion cohort reported here, only one other human study has been published using αCSF1R and a CD40a combination [17]. In the report by Machiels et al., patients with advanced solid tumors treated with αCSF1R and CD40a, the best overall response was SD in 40.5% of patients [17]. This experience similarly does not reflect activity seen in pre-clinical models.

We performed correlative studies on patient material from the melanoma cohort in the phase 1b trial. Non-classical monocytes, believed to be the progenitors of immune suppressive macrophages, were reduced after treatment [27]. This suggests adequate target binding and pharmacodynamic effects of cabiralizumab in the circulation, consistent with other studies reporting pharmacodynamics of CSF1R blockade [13, 28, 29]. We also observed a treatment-induced increase in DCs consistent with activation of CD40 by sotigalimab, although this did not reach statistical significance [30]. The reduction in circulating B-cells is likely also an effect of sotigalimab since similar observations have been made with CD40a monotherapy [31, 32]. Correlative studies reported by Machiels et al. were similar to ours, with reduction in peripheral blood non-classical monocytes (CD14dim CD16bright) and B-cells. Patients in our study who were on treatment for a longer period of time due to clinical benefit had higher expression of CD40 in B-cells and classical monocytes and higher expression of CD86 in DCs. However, we did not see a significant increase in CD8 + T-cells on-treatment compared to baseline, which could partially explain the insufficient anti-tumor activity.

In preclinical models performed by our groups and others, elimination of TAMs by CSF1R blockade alone did not result in substantial inhibition of tumor growth [8, 9, 33]. Other drugs are therefore needed to augment CSF1R blockade, including CD40a. Given the findings that anti-tumoral responses by CD40a and CSF1R blockade are partly dependent on T-cells, we elected to add αPD-1 to our current studies. The lack of increase in circulating CD8 + T-cells in our human studies suggests that alternative modalities are needed for T-cell priming and proliferation.

Although our human correlative studies showed a reduction in non-classical monocytes and an increase of DCs, this was insufficient to translate into clinical and radiographic responses. Due to the limited activity in humans despite the murine data supporting the triple combination, we questioned whether the αCSF1R dose might negatively impact the response and returned to the mouse models to understand the effects of higher versus lower doses of αCSF1R. We showed that a higher αCSF1R dose had clear detrimental effects on tumor growth and animal survival, accompanied by a more immunosuppressive TAM profile at the gene and protein expression level in the melanoma cell line YUMMER1.7.

Specifically, single cell gene expression analyses revealed that TAMs from mice that received higher αCSF1R dose had abundant expression of genes seen with an immune suppressive TAM phenotype *(Lgals1, CD36, Ctsk, Spp1, and Arg1)*. Plasma from the group of mice treated with the higher αCSF1R dose had increased levels of CCL3 and CXCL2. The upregulation of CCL3 and CXCL2 with the higher αCSF1R dose was supported by scRNAseq data in the immune suppressive TAM cluster. Both CCL3 and CXCL2 have been implicated in MDSC recruitment. Immune suppressive effects of increased CCL3 have been observed in melanoma and higher levels of CCR5 ligands, CCL3, CCL4 and CCL5, correlated with accumulation of CCR5 + MDSCs in melanoma lesions and tumor progression. CCR5 + MDSCs exhibit a stronger immune suppressive profile than CCR5- MDSCs. Blocking the interaction between CCR5 and its ligands improved survival and enhanced efficacy of αPD1 blockade in a melanoma mouse model [34]. Our results suggest that higher doses of αCSF1R may lead to an immune suppressive environment via upregulation of CCL3 and CXCL2. MDSCs comprise neutrophils and monocytes and can be grouped into two main types based on their origin, monocytic MDSC (M-MDSC) and granulocytic/ polymorphonuclear MDSC (PMN-MDSC) [35]. In our plasma analysis we observed that treated mice exhibited increased expression of cytokines and chemokines that attract neutrophils. This was supported by our scRNAseq analysis in which we showed an increase in neutrophils after treatment. Signaling through CXCL1 and CXCL2 is mainly responsible for recruitment of immune suppressive tumor associated neutrophils as well as PMN-MDSC [35]. Hence, the possible involvement of neutrophils in an immune suppressive tumor environment cannot be ruled out.

Higher doses of αCSF1R also paradoxically resulted in increased CD163 + positive macrophages in our murine model. Preclinical work reported by Hoves et al. compared the effects of weekly TAM depletion to one dose or two doses of αCSF1R and CD40a. Survival was highest in the group treated only once, followed by the group treated twice, while the worst survival was seen in the group treated weekly [9]. The authors reasoned that the anti-tumoral effect of the αCSF1R and CD40a combination might not be due to constant depletion of TAMs and that an intermittent or less frequent dosing in which recovery of TAMs followed by TAM re-activation might be more favorable. Other preclinical studies have revealed possible mechanisms that might explain why CSF1R blockade therapy fails, including an influx of regulatory T-cells, tumor-promoting granulocytes, TAMs resistant to CSF1R and aberrant signaling of PI3K that is downstream of CSF1R signaling [36–39] .

When we evaluated dosing implications of anti-CSF1R in the parental, less immunogenic cell line, YUMM1.7 we did not see any tumor regression nor difference in survival between lower and higher anti-CSF1R dosing. In contrast to YUMMER1.7, YUMM1.7, lacks the UV induced somatic mutations and has a lower T-cell infiltration which could be necessary for an immune therapy response [19]. This suggests that macrophage modulation in a tumor lacking sufficient T-cell infiltration might not be sufficient to induce an anti-tumor response.

Emactuzumab (αCSF1R antibody) and selicrelumab (CD40 agonist) were studied in a dose escalation study by Machiels et al. [17]. In the cohorts that received the higher doses of emactuzumab and selicrelumab, a more noticeable reduction in CD8 + T-cells was observed. These studies support the observations reported here, that higher doses of αCSF1R is not necessarily better and that further optimization of CSF1R dosing and scheduling is warranted prior to embarking on additional human studies.

This clinical trial was preceded by a phase 2 trial of sotigalimab and nivolumab (without cabiralizumab) in melanoma patients with anti-PD-1 resistant/refractory disease, and a subset of patients had durable responses [16]. Although cross-trial comparisons cannot be made, it appears that the addition of cabiralizumab was not beneficial. The dose of cabiralizumab selected for these studies was based on previous phase I/II trials of cabiralizumab alone and with αPD-1 [26]. The maximum tolerated dose is typically used to determine the RP2D. Our pre-clinical studies demonstrating superior anti-tumor activity with lower dose of αCSF1R combined with the disappointing activity in the human trial raise the question of whether clinical trial designs should be modified to better assess pharmacodynamic effects on tumor and tumor-infiltrating cells to identify the RP2D, rather than solely basing the RP2D on tolerability and safety.

## Conclusions

Cabiralizumab at the RP2D, in combination with sotigalimab and nivolumab had limited anti-tumor activity in humans. CSF1R dosing studies in a partially immunogenic mouse model indicate that higher dose of αCSF1R are inferior, and the suppressive TAM phenotype observed with higher doses might impede the desired response. Optimization of the dosing of cabiralizumab is necessary to further assess its clinical potential in combination with other macrophage and T-cell modulating drugs in a difficult-to treat patient population whose therapeutic options are limited.

## Methods

### Phase I/Ib study

This was a phase I/Ib study of sotigalimab (CD40a) in combination with nivolumab (αPD-1) and cabiralizumab (αCSF1R) in patients with advanced solid tumors whose disease progressed on αPD-(L)1 therapy. The primary objectives of the phase I dose escalation portion were to determine safety of the doublet (cabiralizumab plus sotigalimab) and triplet (doublet plus nivolumab) in patients with advanced melanoma, NSCLC or RCC as well as the RP2D. Sotigalimab was dose escalated from 0.03 to 0.1 to 0.3 mg/kg intravenously (IV) using a 3 + 3 design, in combination with fixed doses of cabiralizumab IV at 4 mg/kg plus or minus nivolumab IV at 240 mg every 2 weeks. The RP2D of the triplet was determined to be sotigalimab 0.3 mg/kg in combination with cabiralizumab 4 mg/kg and nivolumab 240 mg IV every 2 weeks. The phase I results have been published [18]. A phase Ib dose expansion portion was conducted for melanoma patients and is reported here. Patient material from phase Ib was analyzed for pharmacodynamic studies reported here.

### Statistical clinical trial design for phase Ib

Eligible patients for the phase Ib dose expansion portion were treated in two disease-specific cohorts, advanced melanoma (reported here, Table 1) or NSCLC. Patients were required to have biopsy-proven disease with radiographic and/or clinical progression on αPD-(L)1 without intervening therapy. Eligibility criteria included age ≥ 18, ECOG performance status 0–1, life expectancy > 6 months, and normal organ function. Patients with melanoma were included irrespective of BRAF status. Any number of prior therapies was allowed, however patients treated with prior αCSF1R and/or CD40a therapies were excluded. A detailed list of eligibility criteria can be found in the study protocol (Related files).

### Treatment and assessments for phase Ib

Prophylactic medications administered 30 min before treatment included diphenhydramine, famotidine, ibuprofen and acetaminophen. Nivolumab, cabiralizumab, and sotigalimab were administered sequentially over 30, 30, and 60 min, respectively, with 30 min breaks in between. Treatment was administered every 14 days until disease progression, intolerable toxicity, or consent withdrawal. Radiographic assessments including body CT or PET CT and MRI brain were performed at baseline, every 8 weeks for 4 months, and every 12 weeks thereafter. Treatment beyond progression was allowed if clinical benefit was derived as determined by the treating investigator.

### Objectives for phase Ib

The primary objectives were to determine the ORR using RECIST v1.1 to sotigalimab in combination with cabiralizumab and nivolumab in patients with advanced melanoma and to evaluate the safety and tolerability of the regimen. Secondary objectives were to determine PFS and OS and to assess the association of selected biomarker and clinical efficacy measures using pre-treatment and on-treatment tumor biopsies. Exploratory objectives were to identify immune correlates that are associated with clinical response or resistance to the combination.

### Statistical methods for phase Ib

Simon’s two-stage design was used. The null hypothesis that the true response rate to a regimen with minimal activity is 10% was tested against a one-sided alternative. In the first stage, 13 patients in each disease cohort were planned for accrual. If there were 1 or fewer responses in these 13 patients, then enrollment in that disease cohort would be stopped. Otherwise, 21 additional patients would be accrued for a total of 34 patients per disease cohort. The null hypothesis would be rejected if 6 or more responses are observed in 34 patients. This design yields a type I error rate of 0.1 and a power of 80% when the true response rate is 25%.

### CyTOF

PBMCs were collected at three time points: C1D1, C1D2 and C2D1. CyTOF was performed on frozen PBMCs as described [40]. In brief, cells were barcoded with anti-CD45 antibodies conjugated to unique metal isotopes before pooling samples together to limit batch effects. Cells were stained with Cell-ID Intercalator-103Rh viability marker and a panel of 33 metal-conjugated antibodies (Supplementary Table 2). Cells were spiked with EQ 4 element beads (Fluidigm) and acquired in Helios mass cytometry system (Fluidigm). Bead-based normalization and debarcoding were performed. We processed 37 samples (3 time points from 11 patients and 2 time points from 2 patients). Left-over beads, debris, dead cells, and doublets were eliminated leaving singlet CD45 + cells [41]. Gating was done in Cytobank. The gates of various cell populations are shown in dot plots (Supplementary Fig. 4 and Supplementary Table 3) [41]. Main cell populations and their respective subpopulations were analyzed as percentage of CD45 + CD66b- cells at timepoints C1D1, C1D2 and C2D1 for treatment related changes. Ratios of different cell populations between timepoints C1D1-C2D1 and C1D1-C1D2 were analyzed for changes associated with duration of time on trial (< 200 days on trial vs > 200 days on trial) and best response (PD vs SD/PR). Ratios of mean expression of individual markers within a cell population between timepoints C1D1-C2D1 and C1D1-C1D2 were analyzed for changes associated with duration of time on trial (< 200 days on trial vs > 200 days on trial) and best response (PD vs SD/PR).

### Statistical methods for correlative studies from phase Ib

Paired t-tests were used to analyze percentage of cell populations differences between different time points (C1D1, C1D2 and C2D1). Unpaired t-tests were used to analyze ratios of different cell populations or individual markers’ association with duration of time on trial and best response. A *P* value ≤ 0.05 was considered statistically significant.

### In vivo melanoma models

2 × 10^6^ YUMMER1.7 cells or 5 × 10^5^ YUMM1.7 cells (kindly gifted from Dr. Marcus Bosenberg, Yale University; RRID:CVCL_A2AX and RRID:CVCL_JK16) were subcutaneously injected into the left flank of 8–9 week-old C57Bl6 male mice. YUMM1.7 is a Braf^V600E^ /Pten^−/−^, Cdkn2a^−/−^ cell line. YUMMER1.7 is a UV irradiated derivative of YUMM1.7 carrying a higher number of somatic mutations in addition to the three driver mutations: Braf^V600E^, Pten^−/−^ and Cdkn2a^−/−^ [19]. Seven days after tumor cell injection, 200 μg αPD1 (Bio X Cell, Clone RMP1-14), 200 μg αCD40 (Bio X Cell, Clone: FGK4.5/ FGK45) and 200 μg or 400 μg αCSF1R (Bristol Myers Squibb, mG1D265A) were injected IP twice a week for a total of five treatments. 200 μg of αCSF1R was the dose initially chosen (“lower dose”). We arbitrarily chose to double the initial dose to study “higher dose”. Control mice received PBS. Tumors were measured with a digital caliper and tumor volume estimated with the ellipsoid volume calculation formula. Endpoint was defined as the time until tumors reached 1000 mm^3^.

### Cytokine/chemokine profiling for murine experiments

Whole blood was collected from control or treated mice 24 h after the first treatment in EDTA tubes. Plasma was analyzed for cytokine/chemokine expression (31-plex Mouse Cytokine / Chemokine Array, cat# MD31, Eve Technologies). Cytokine/chemokine levels (pg/ml) were analyzed in R Studio (version 3.6.2) or GraphPad Prism 9.

### Immunohistochemistry of murine tumors

YUMMER1.7 tissues collected at endpoint were fixed in 10% neutral-buffered formalin overnight and embedded in paraffin by the Yale Pathology department Histology core. 5 μm FFPE tumor sections were stained with antibodies towards CD3 (cat# CP215, Biocare Medical), CD8 (clone 4SM15, eBioscience), CD163 (clone M-96, Santa Cruz), and CD68 (cat# ABIN3044428, Antibodies online). Three random intra tumoral regions were selected and positive cells were manually counted. The values in treated groups relative to controls were plotted in GraphPrism 9.

### scRNA sequencing of murine tumors

Groups of three mice were treated IP on day 7 post YUMMER1.7 cell injection. On day 8, tumors were minced in RPMI with 2% FBS, incubated with 0.1 mg/ml collagenase and DNase I for 30 min at 37ºC. Cells were filtered through 70 μM filters to obtain single cell suspension, washed with RPMI + 10% FBS and pelleted by centrifugation at 1750 rpm for 5 min and resuspended in RPMI + 20% FBS. For sorting, cells were incubated for 30 min at 4ºC with fluorophore-conjugated antibodies. Samples were sorted into three populations: tumor cells (CD45- cells), myeloid cells (CD45 + CD3-, CD19-, NK1.1- cells) and CD45 + , CD3 + , CD19 + , NK1.1 + (B cells, T-cells and NK cells). Antibodies used for cell sorting were anti-CD45 (clone 30-F11, BD Biosciences), anti-CD3 (clone 17A2, BD Bioscience), anti-CD19 (clone 1D3, BD Biosciences) and anti-NK1.1(clone PK136, BD Biosciences). For live/dead staining, AmCyan Kit (Thermo Fisher Scientific) was used. Sorted cell subsets (BD FACSAriaII) were combined at a ratio of 50% tumor cells, 30% B cells, T-cells and NK cells and 20% myeloid cells. Library preparation was performed for scRNA-seq using the 3′ transcriptome kit (10 × Genomics) according to the manufacturer’s instructions. cDNA libraries were sequenced on a NovaSeq instrument (Illumina) at the Yale Center for Genome Analysis and the aligned reads were mapped to the mouse reference transcriptome (mm9). Digital count matrices were analyzed to identify cell types using R Studio (version 4.1.2) and the package Seurat v.4.0.5. To remove low quality cells following thresholds were applied: > 500 nUMI, > 250 genes, > 0.8 log10GeneperUMI and < 0.2 mitochondrial gene ratio and only genes expressed in 10 or more cells. Data was normalized and integrated using the “sctransform” method. Principal component (PC) scores from the first 40 PCs were used for clustering. For dimension reduction, Uniform Manifold Approximation and Projection (UMAP) was used with a resolution of 0.6. Distinct cell types were generated using the function “FindMarkers to determine genes that are differentially expressed between each cluster. For this, each cluster (cluster 0) was compared to all the remaining clusters (clusters 1–18). This generated a list of genes for each cluster including an average log fold change (positive value means that the gene is more highly expressed in the cluster being compared to all other clusters), pct.1 informs of percentage of cells in which the gene is detected in the first group (e.g. cluster 0), pct.2, informs of the percentage of cells where the gene is detected in the second group (e.g. clusters 1–18), and an adjusted p-value based on Bonferroni correction using all genes in the dataset is generated.

### Statistical methods for murine studies

Log-rank statistics were used for survival analysis. Mann–Whitney test was used to analyze difference in expression levels of cytokine/chemokine between treatment groups. Unpaired t-test was used to analyze the difference in positive cells stained for IHC analysis in tumors. Wilcoxon Rank Sum test was used for scRNAseq data to identify distinct cell types. A *P* value ≤ 0.05 was considered statistically significant.

### Supplementary Information

**Additional file 1:** **Supplementary figure 1.** Changes in peripheral blood mononuclear cell (PBMC) populations. **Supplementary figure 2.** Survival in YUMM1.7 melanoma model treated with lower vs. higher dose of anti-CSF1R. **Supplementary figure 3.** Higher dose of anti-CSF1R results in increased CCL3 and CXCL2 expression in TAM-IIs. **Supplementary figure 4.** Marker expression in key cell populations from CyTOF depicted via dot plot. **Supplementary Table 1.** Genes expressed in tumor associated macrophage (TAM) -I and TAM-II clusters. **Supplementary Table 2.** Antibodies used for CyTOF analysis. **Supplementary Table 3.** Marker expression in key PBMC populations from CyTOF.

## Data Availability

The datasets used and/or analyzed during the current study are available from the corresponding author on reasonable request.

## Abbreviations

ARG1: Arginase 1
AST: Aspartate aminotransferase
C1D1: Cycle 1 day 1
CD40a: CD40 agonist
CPK: Creatine phosphokinase
CSF1R: Colony-stimulating factor 1 receptor
CTLA-4: Cytotoxic T-lymphocyte associated protein-4
CyTOF: Cytometry by time of flight
DSP: Digital spatial profiling
ECOG: Eastern Cooperative Oncology Group
FFPE: Formalin fixed paraffin embedded
IP: Intraperitoneal
IV: Intravenous
MDSCs: Myeloid-derived suppressor cells
NSCLC: Non-small cell lung cancer
ORR: Objective response rate
OS: Overall survival
PBMCs: Peripheral blood mononuclear cells
PC: Principal component
PD: Progressive disease
PD-1: Programmed cell death protein -1
PD-L1: Programmed cell death ligand 1
PFS: Progression free survival
PR: Partial response
RCC: Renal cell carcinoma
RP2D: Recommended phase 2 dose
scRNAseq: Single cell RNA sequencing
SD: Stable disease
TAMs: Tumor associated macrophages
TILs: Tumor infiltrating lymphocytes
TMA: Tissue microarray
TME: Tumor microenvironment
TRAEs: Treatment-related adverse events
tSNE: T-distributed stochastic neighbor embedding
YUMMER: Yale University Mouse Melanoma Exposed to Radiation
