# Supplementary material for: A bedside to bench study of anti-PD-1, anti-CD40, and anti-CSF1R indicates that more is not necessarily better
Source: Mol Cancer. 2023 Nov 14;22:182. doi: 10.1186/s12943-023-01884-x (PMC10644655; doi:10.1186/s12943-023-01884-x)
Supplement: Supplementary file 1 — Additional file 1: Supplementary figure 1. Changes in peripheral blood mononuclear cell (PBMC) populations. Supplementary figure 2. Survival in YUMM1.7 melanoma model treated with lower vs. higher dose of anti-CSF1R. Supplementary figure 3. Higher dose of anti-CSF1R results in increased CCL3 and CXCL2 expression in TAM-IIs. Supplementary figure 4. Marker expression in key cell populations from CyTOF depicted via dot plot. Supplementary Table 1. Genes expressed in tumor associated macrophage (TAM) -I and TAM-II clusters. Supplementary Table 2. Antibodies used for CyTOF analysis. Supplementary Table 3. Marker expression in key PBMC populations from CyTOF. [file 12943_2023_1884_MOESM1_ESM.docx]

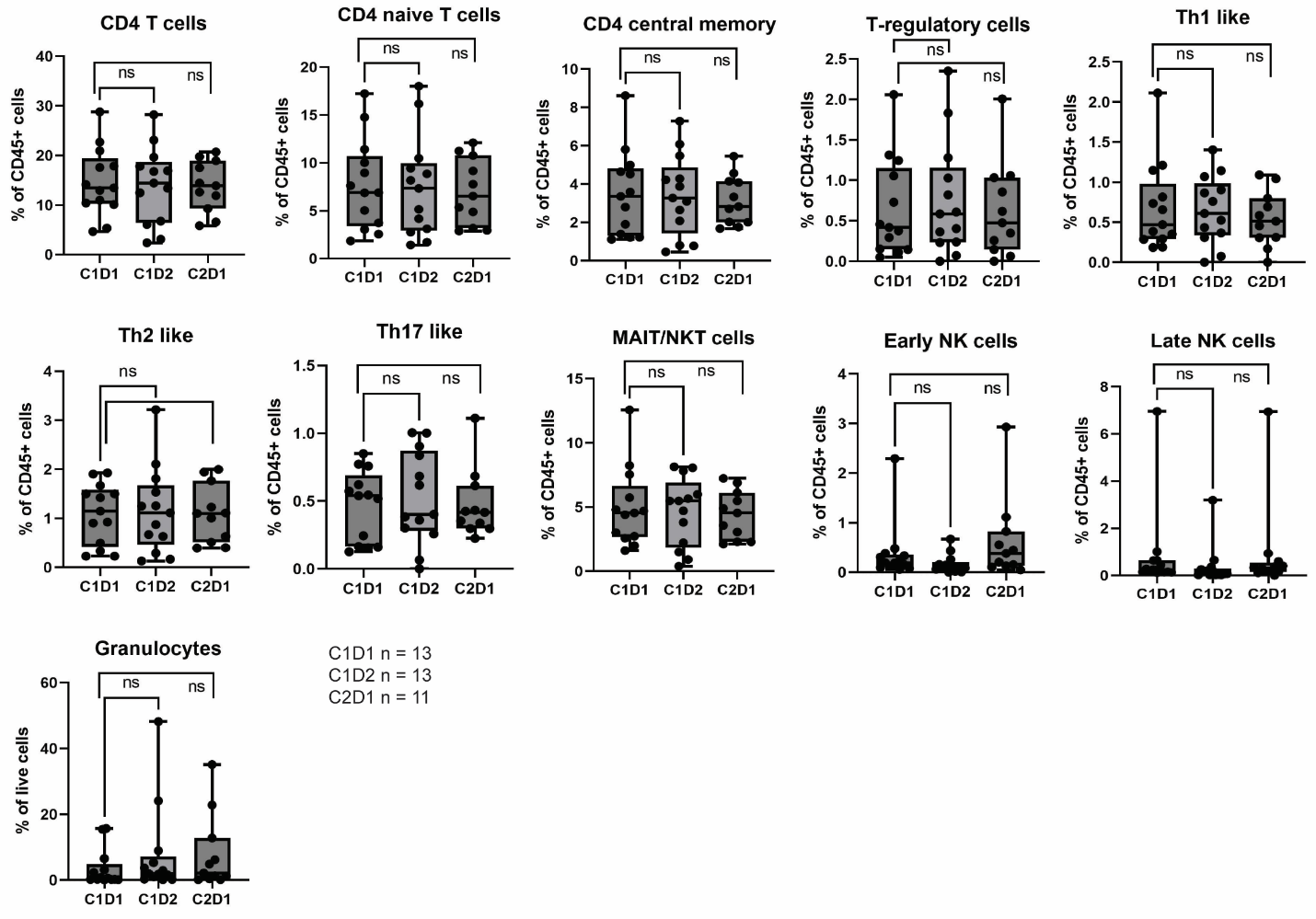


**Supplementary figure 1.** Changes in peripheral blood mononuclear cell (PBMC) populations. PBMC changes in patients treated with sotigalimab, cabiralizumab and nivolumab at cycle 1 day 1 (C1D1), cycle 1 day 2 (C1D2) and cycle 2 day 1 (C2D1). ns= not significant


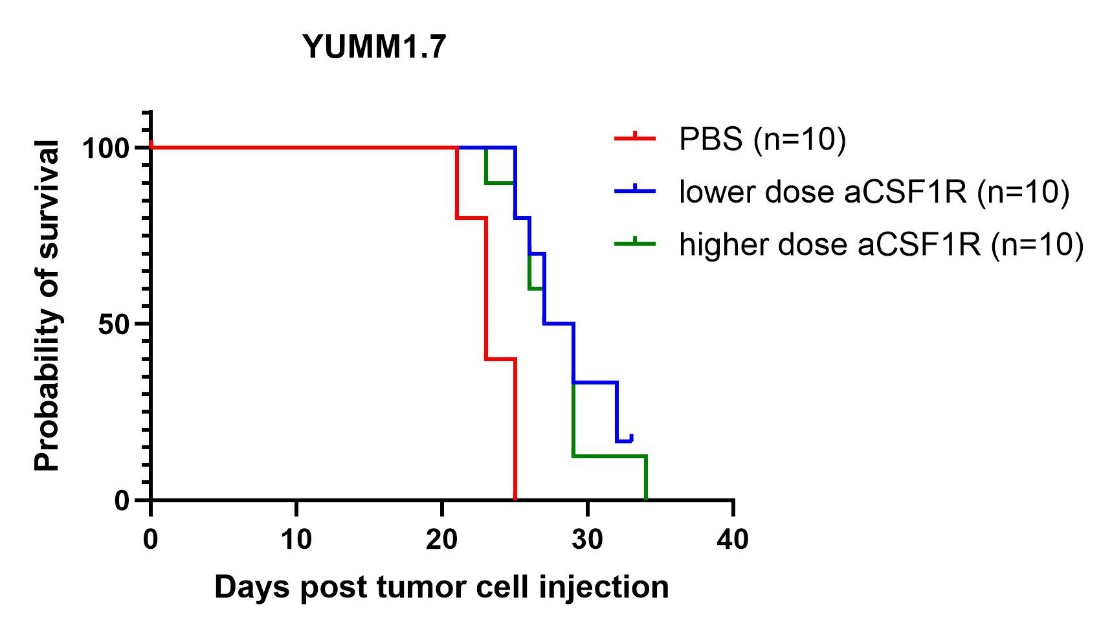


**Supplementary figure 2.** Survival in YUMM1.7 melanoma model treated with lower vs. higher dose of anti-CSF1R. Survival curve of untreated YUMM1.7 melanoma mice and YUMM1.7 melanoma mice treated anti-PD1, anti-CD40, and either lower (200ug) or higher (400ug) dose of anti-CSF1R. p =0.7.


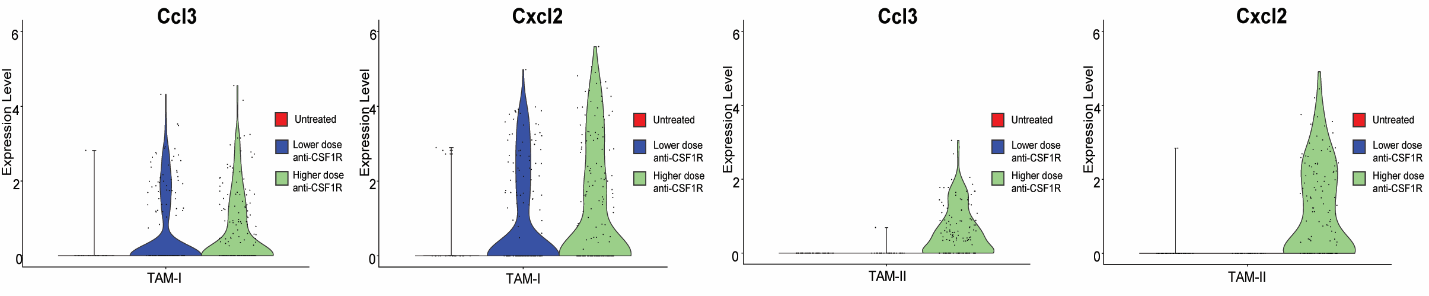


**Supplementary figure 3.**  Higher dose of anti-CSF1R results in increased CCL3 and CXCL2 expression in TAM-IIs. Expression of CCL3 and CCL2 detected via single cell RNA sequencing in TAM-I and TAM-II from untreated YUMMER1.7 mice or mice treated with anti-PD1, anti-CD40, and either lower (200 ug) or higher (400 ug) dose anti-CSF1R.


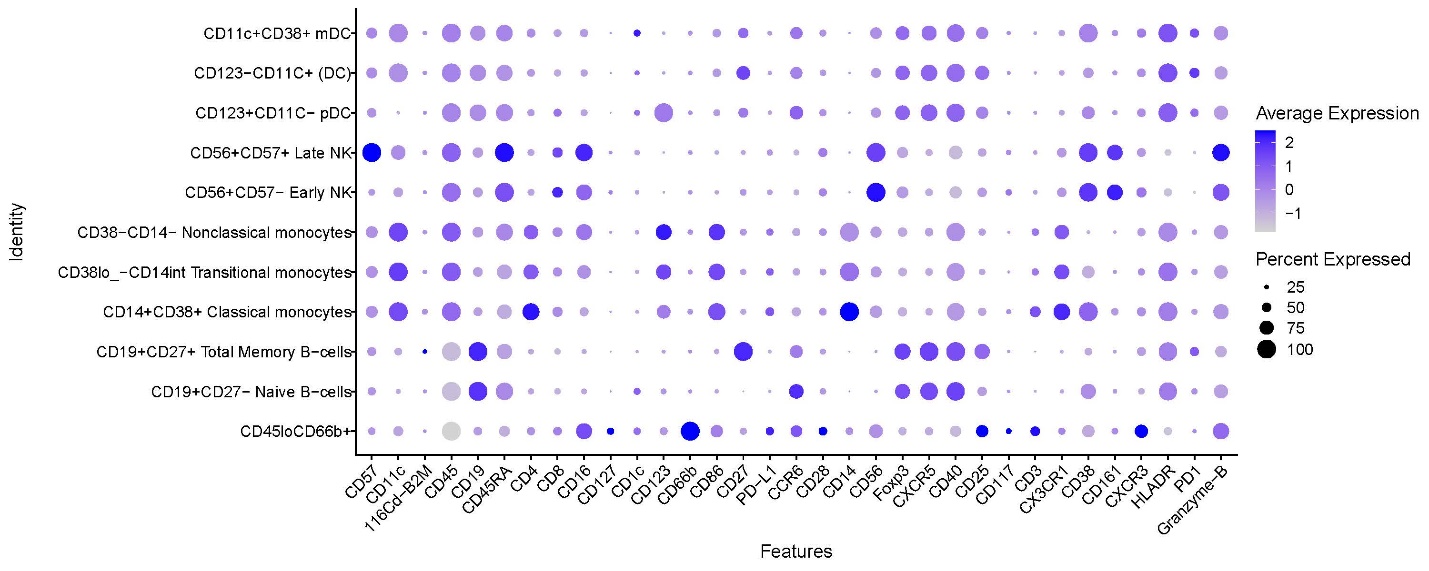

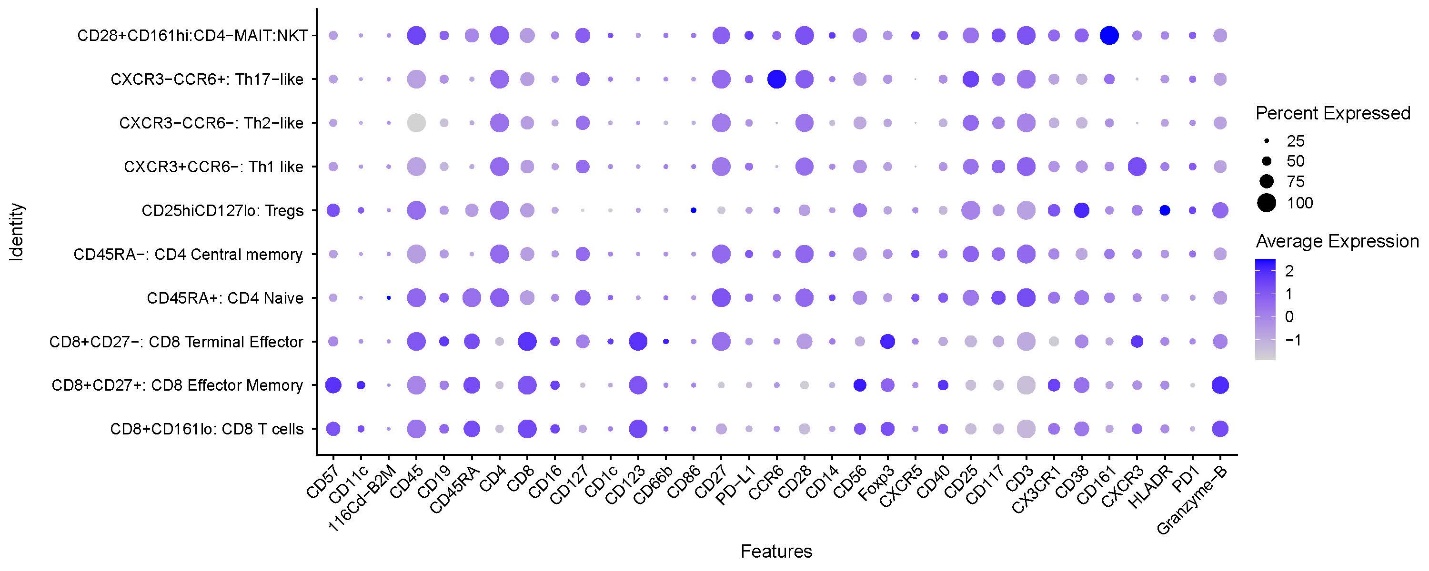
**Supplementary figure 4**. Marker expression in key cell populations from CyTOF depicted via dot plot. Markers used for gating are indicated left of cell type label (y-axis). A. Main cell populations. B. Gates in T cell populations

Supplementary Table 1. Genes expressed in tumor associated macrophage (TAM) -I and TAM-II clusters.

| **TAM -I** | **Gene** | **Avg_log2FC** | **Pct.1** | **Pct.2** | **P_val_adj** | **Description** | **DOI** |
| --- | --- | --- | --- | --- | --- | --- | --- |
| 1 | Smpdl3b | 1.627526 | 0.416 | 0.018 | 5.96E-301 | Suppressive | 10.1016/j.celrep.2015.05.006 |
| 2 | Lyz2 | 3.497195 | 0.924 | 0.176 | 2.01E-298 | Inflammatory | 10.1016/j.immuni.2017.04.018 |
| 3 | Nos2 | 2.448962 | 0.382 | 0.014 | 1.24E-286 | Inflammatory | 10.1111/febs.15715 |
| 4 | Pla2g7 | 2.28719 | 0.811 | 0.126 | 1.99E-272 | Suppressive | 10.1038/s41420-022-00831-x. |
| 5 | Clec4e | 2.091216 | 0.684 | 0.079 | 2.74E-272 | Inflammatory | 10.1016/j.kint.2016.10.020 |
| 6 | Lst1 | 2.179043 | 0.845 | 0.156 | 1.48E-261 | Inflammatory | 10.3389/fimmu.202 |
| 7 | Ifitm3 | 2.543081 | 0.966 | 0.249 | 9.21E-245 | Inflammatory | 10.1158/0008-5472.CAN-21-0101 |
| 8 | Klra2 | 1.225064 | 0.413 | 0.026 | 5.18E-244 | NA |  |
| 9 | Ly6c2 | 2.898478 | 0.732 | 0.115 | 1.16E-243 | ? | 10.26508/lsa.202000935 |
| 10 | Il1b | 1.73419 | 0.837 | 0.146 | 6.86E-240 | Inflammatory | 10.1016/j.joca.2021.09.003. |
| 11 | Ifi205 | 1.204324 | 0.353 | 0.018 | 1.78E-232 | NA |  |
| 12 | Fpr2 | 1.514532 | 0.453 | 0.034 | 1.09E-231 | Suppressive | 10.1186/s13048-021-00932-8 |
| 13 | Fcer1g | 2.452005 | 0.974 | 0.28 | 1.76E-230 | Both | 10.1155/2022/7153491 |
| 14 | Ctsc | 2.24742 | 0.826 | 0.168 | 3.23E-230 | Inflammatory? | 10.1016/j.yexcr.2019.06.017 |
| 15 | Mcemp1 | 1.573712 | 0.645 | 0.083 | 6.73E-226 | Inflammatory? | 0.1155/2022/8167496 |
| 16 | Clec4a1 | 1.017286 | 0.366 | 0.022 | 3.88E-223 | Inflammatory | 10.1016/j.bbrc.2016.10.030 |
| 17 | Ifi211 | 1.45403 | 0.597 | 0.077 | 3.43E-220 | NA |  |
| 18 | Cebpb | 2.291618 | 0.947 | 0.286 | 2.20E-214 | Inflammatory | 10.3389/fimmu.2018.02515 |
| 19 | Prdx5 | 2.802644 | 0.955 | 0.331 | 5.41E-213 | Inflammatory | 10.1016/j.chembiol.2018.02.006 |
| 20 | Calhm6 | 1.559151 | 0.424 | 0.035 | 2.53E-210 | Inflammatory | 10.1016/j.gene.2016.10.029, 10.1186/s12879-021-05888-0 |
| **TAM - II** |  |  |  |  |  |  |  |
| 1 | Gpnmb | 3.572411 | 0.541 | 0.039 | 7.45E-224 | Suppressive | 10.1038/s41423-020-0501-0 |
| 2 | Myof | 0.968664 | 0.573 | 0.043 | 3.69E-221 | NA |  |
| 3 | F7 | 0.706148 | 0.362 | 0.016 | 2.58E-198 | ? |  |
| 4 | Lgals1 | 3.351572 | 0.972 | 0.225 | 5.80E-190 | Suppressive | 10.1007/s00262-021-03139-4. |
| 5 | Cd36 | 1.347046 | 0.353 | 0.016 | 7.60E-190 | Supressive | 10.1158/0008-5472.CAN-19-2994 |
| 6 | Ctsk | 0.70497 | 0.307 | 0.011 | 2.01E-186 | Supressive | 10.1038/s41418-019-0312-y |
| 7 | Emp1 | 1.467068 | 0.546 | 0.048 | 6.04E-186 | Both | 10.3892/ol.2020.11841 |
| 8 | Vat1 | 0.921242 | 0.518 | 0.045 | 1.05E-175 | ? |  |
| 9 | Spp1 | 4.188225 | 0.606 | 0.069 | 4.83E-175 | Supressive | 10.1136/jitc-2021-002624 |
| 10 | S100a4 | 2.577351 | 0.885 | 0.152 | 1.82E-172 | Supressive | 10.1136/jitc-2021-002548 |
| 11 | Tnfsf13 | 1.251132 | 0.55 | 0.058 | 5.72E-164 | Supressive | 10.1007/s10059-011-1040-4 |
| 12 | Rab7b | 0.588573 | 0.468 | 0.038 | 2.95E-163 | NA |  |
| 13 | Arg1 | 2.913238 | 0.44 | 0.034 | 5.43E-163 | Supressive |  |
| 14 | Apoc2 | 2.593423 | 0.564 | 0.063 | 1.82E-159 | NA |  |
| 15 | Dnase1l1 | 0.503234 | 0.495 | 0.045 | 2.65E-159 | NA |  |
| 16 | Anxa1 | 2.035691 | 0.619 | 0.075 | 1.66E-155 | Supressive | 10.3390/ijms23158256. |
| 17 | Ms4a7 | 1.729835 | 0.518 | 0.052 | 2.70E-154 | NA |  |
| 18 | Msr1 | 1.017224 | 0.509 | 0.05 | 2.69E-153 | Supressive | 10.1016/j.intimp.2022.109217 |
| 19 | Pdpn | 0.391901 | 0.257 | 0.01 | 6.00E-152 | Inflammatory | 10.1182/blood.2019001388. |
| 20 | Fam20c | 0.625589 | 0.45 | 0.039 | 2.55E-149 | Supressive? | 10.1042/BSR20201920. |

Average log fold change: positive value means that the gene is more highly expressed in the cluster being compared to all other clusters (here in TAM-1 or TAM-II compared to other clusters). Pct.1 represents percentage of cells in which the gene is detected in the first group (TAM-I or TAM-II) and Pct.2 represents percentage of cells in which the gene is detected in the second group (all other clusters besides TAM-1 or TAM-II). Adjusted p value: based on Bonferroni correction using all genes in the data set.

Supplementary Table 2. Antibodies used for CyTOF analysis.

| **Target** | **Isotype** |
| --- | --- |
| CD45 | 89 Y |
| CD57 | 113 In |
| CD11c | 115 In |
| CD19 | 142 Nd |
| CD45RA | 143 Nd |
| CD4 | 145 Nd |
| CD8 | 146 Nd |
| CD16 | 148 Nd |
| CD127 | 149 Sm |
| CD1c | 150 Nd |
| CD123 | 151 Eu |
| CD66b | 152 Sm |
| CD86 | 154 Sm |
| CD27 | 155 Gd |
| PD-L1 | 156 Gd |
| CCR6/CD196 | 158 Gd |
| CD28 | 159 Tb |
| CD14 | 160 Gd |
| CD56 | 161 Dy |
| FoxP3 | 162 Dy |
| CXCR5/CD185 | 163 Dy |
| CD40 | 164 Dy |
| CD25 | 166 Er |
| CD117 | 167 Er |
| CD3 | 168 Er |
| CX3CR1 | 169 Tm |
| CD38 | 170 Er |
| CD161 | 171 Yb |
| CXCR3 | 173 Yb |
| HLADR | 174 Yb |
| PD-1/CD279 | 175 Lu |
| Granzyme B | 176 Yb |

Supplementary Table 3. Marker expression in key PBMC populations from CyTOF.

**Cell population Markers**

Granulocytes CD45lo,CD66b+

Total B cells CD45+,CD66b-, CD56-,CD14-, CD3-,CD19+

Naïve B cells CD45+,CD66b-, CD56-,CD14-, CD3-,CD19+, CD27-

Memory B cells CD45+,CD66b-, CD56-,CD14-, CD3-,CD19+, CD27+

Total monocytes CD45+,CD66b-, CD19-, CD3-,CD56-, CD11c+,HLADR+, CD14+,CD11c+

Classical monocytes CD45+,CD66b-, CD19-, CD3-,CD56-, CD11c+,HLADR+, CD14+,CD38+

Non-classical monocytes CD45+,CD66b-, CD19-, CD3-,CD56-, CD11c+,HLADR+, CD14-,CD38-

Intermediate monocytes CD45+,CD66b-, CD19-, CD3-,CD56-, CD11c+,HLADR+,CD14int,CD38lo

Total NKs CD45+,CD19-, CD3-, CD14-, CD45RA+,CD123-, CD56+

Early NKs CD45+,CD19-, CD3-, CD14-, CD45RA+,CD123-, CD56+, CD57-

Late NKs CD45+,CD19-, CD3-, CD14-, CD45RA+,CD123-, CD56+, CD57+

Total DCs CD45+,CD19-,CD3-,CD14-,HLA-DR+

plasmacytoid DCs CD45+,CD19-,CD3-,CD14-,HLA-DR+,CD123+,CD11c-

myeloid DCs CD45+,CD19-,CD3-,CD14-,HLA-DR+,CD123-,CD11c+, CD38+

MAIT/NKT cells CD45+,CD19-,CD3+,CD4-,CD14-CD11c,CD28+,CD161hi

Total T cells CD45+,CD66b-,CD19-,CD14-,CD11c-,CD3+

CD8 T cells CD45+,CD66b-,CD19-,CD14-,CD11c-,CD3+, CD4-, CD8+, CD161lo

CD8 effector memory CD45+,CD66b-,CD19-,CD14-,CD11c-,CD3+, CD4-, CD8+, CD161lo,CD27+

CD8 terminal effector like CD45+,CD66b-,CD19-,CD14-,CD11c-,CD3+, CD4-, CD8+, CD161lo,CD27-

CD4 T cells CD45+,CD66b-,CD19-,CD14-,CD11c-,CD3+, CD8-,CD4+

CD4 Naïve CD45+,CD66b-,CD19-,CD14-,CD11c-,CD3+, CD8-,CD4+,CD27hi, CD45RA+

CD4 central memory CD45+,CD66b-,CD19-,CD14-,CD11c-,CD3+, CD8-,CD4+,CD27hi,CD45RA-

T regulatory cells CD45+,CD66b-,CD19-,CD14-,CD11c-,CD3+, CD8-,CD4+, CD27-, CD25hi,CD127lo

Terminal effector CD45+,CD66b-,CD19-,CD14-,CD11c-,CD3+, CD8-,CD4+,CD27lo,CD45RA-

Th1 like CD45+,CD66b-,CD19-,CD14-,CD11c-,CD3+, CD8-,CD4+,CXCR5-, CD45RA-,CD27+,CXCR3+,CCR6-

Th2 like CD45+,CD66b-,CD19-,CD14-,CD11c-,CD3+, CD8-,CD4+,CXCR5-, CD45RA-,CD27+, CXCR3-,CCR6-

Th17 like CD45+,CD66b-,CD19-,CD14-,CD11c-,CD3+, CD8-,CD4+,CXCR5-, CD45RA-, CD27+,CXCR3-,CCR6+
